# Variation in the Structure of Bird Nests between Northern Manitoba and Southeastern Ontario

**DOI:** 10.1371/journal.pone.0019086

**Published:** 2011-04-28

**Authors:** Carla A. Crossman, Vanya G. Rohwer, Paul R. Martin

**Affiliations:** 1 Department of Biology, Queen's University, Kingston, Canada; 2 Yanayacu Biological Station, Napo, Ecuador; University of Western Ontario, Canada

## Abstract

Traits that converge in appearance under similar environmental conditions among phylogenetically independent lineages are thought to represent adaptations to local environments. We tested for convergence in nest morphology and composition of birds breeding in two ecologically different locations in Canada: Churchill in northern Manitoba and Elgin in southeastern Ontario. We examined nests from four families of passerine birds (Turdidae: *Turdus*, Parulidae: *Dendroica*, Emberizidae: *Passerculus* and Fringillidae: *Carduelis*) where closely related populations or species breed in both locations. Nests of American Robins, Yellow Warblers, and *Carduelis* finches had heavier nest masses, and tended to have thicker nest-walls, in northern Manitoba compared with conspecifics or congenerics breeding in southeastern Ontario. Together, all species showed evidence for wider internal and external nest-cup diameters in northern Manitoba, while individual species showed varying patterns for internal nest-cup and external nest depths. American Robins, Yellow Warblers, and *Carduelis* finches in northern Manitoba achieved heavier nest masses in different ways. American Robins increased all materials in similar proportions, and Yellow Warblers and Common Redpolls used greater amounts of select materials. While changes in nest composition vary uniquely for each species, the pattern of larger nests in northern Manitoba compared to southeastern Ontario in three of our four phylogenetically-independent comparisons suggests that birds are adapting to similar selective pressures between locations.

## Introduction

Broadly distributed organisms face a myriad of environmental challenges and, in response to such challenges, many possess traits well suited to their local environment [1], [2]. If local selective pressures pose a challenge to many organisms, we may expect particular traits that increase fitness under local conditions to converge in appearance among diverse taxa [3]. When a particular trait converges in appearance in multiple, phylogenetically-independent lineages that experience similar environmental conditions, then the trait is often interpreted as an adaptation to local conditions [3], [4]. Among birds, there are several examples of traits converging under similar environments such as bill morphology [5], song characteristics [6], [7], and body morphology [8]. We might expect further examples of traits that converge in appearance and function under similar environments when variation in these traits confers high fitness advantages, and are thus open to strong directional selection.

The nests of birds are important for successful reproduction because they provide shelter for eggs and nestlings and, in many cases, help incubating and brooding parents conserve energy [9], [10]. One challenge for breeding birds is maintaining warm nest temperatures (36–40°C) during incubation [11], [12]. When ambient temperatures fall outside this range, embryo development slows, or at extreme temperatures, embryos may die. Thus the incubating parents face a trade-off between keeping the eggs and young warm versus foraging for nestlings and self-maintenance.

While ambient temperature may be an important selective factor on nest morphology, many other ecological factors also influence nest morphologies and vary geographically. In windy environments, dense impenetrable nests-walls may be advantageous because they help to minimize convective heat loss [13]. Similarly, wet environments may favor porous nests that absorb little water and dry rapidly [14], and thus minimize the cooling effects of water on eggs, nestlings and adults trying to maintain warm nests. While differences in climate are perhaps the most commonly invoked factors to explain differences in nest morphology, many other factors such as predation, brood parasitism, nest ectoparasitism, and variation in life history strategies (e.g., variation in reproductive effort) may affect nest morphology in birds. For example, differences in nest predator abundance and diversity between breeding sites likely selects for different nest sizes. Using artificial nests, MØller [15] suggested that larger nests suffer higher rates of predation than smaller nests. Similar to nest predation, brood parasites like Brown-headed Cowbirds (*Molothrus ater*) in North America and Common Cuckoos (*Cuculus canorus*) in Europe may preferentially find bulky nests, and thus act as a selective pressure favoring smaller, less conspicuous host nests over time. While at least one study has examined how nest composition may change in response to ectoparasites [16], the influence of nest ectoparasites and varying life history strategies on nest morphologies remain largely unexplored.

Most studies examining geographic variation in bird nests have attributed differences in nest morphology primarily to differences in climate [14], [17], [18]. In most cases, individuals breeding in colder locations built nests that were larger and made of materials that provided better insulation against cold temperatures [14], [17], [18]. One study found differences in nest placement and attachment [13] and attributed these differences to wind and predation pressures from squirrels, and two studies that examined sparrows that often nest on the ground found minimal geographic variation in nest morphologies [19], [20].

Together, these studies suggest that many species of birds have some degree of variation in nest morphology and that local selective pressures (e.g., climate, predation) may cause this variation. While many studies have examined geographic variation in nest morphologies within a single species, few have examined nests from diverse species across a range of habitats [21].

We examined nest morphology (e.g., mass, nest-wall thickness, internal and external nest-cup depth, internal and external nest-cup diameter) and nest composition of four phylogenetically independent lineages of birds to determine if nest morphologies converge under similar breeding conditions. We compared nests of American Robins (*Turdus migratorius*), Yellow Warblers (*Dendroica petechia*), Savannah Sparrows (*Passerculus sandwichensis*), and two species in the genus *Carduelis*, Common Redpolls (*C. flammea*) and American Goldfinches (*C. tristis*), between two breeding locations in Canada: Churchill in northern Manitoba, and Elgin in southeastern Ontario. Pairing *Carduelis* finches was necessary because neither species breeds in both locations; Common Redpolls breed in Churchill and American Goldfinches breed in Elgin. These closely related *Carduelis* finches represent a strong comparison because there is no gene flow between populations that may impede differentiation in nest morphology [22], and both species build cup nests of similar structure. Because all lineages breed in both locations, they provide useful comparisons of how populations may differ in their nest construction in these different breeding environments.

**Figure 1:**
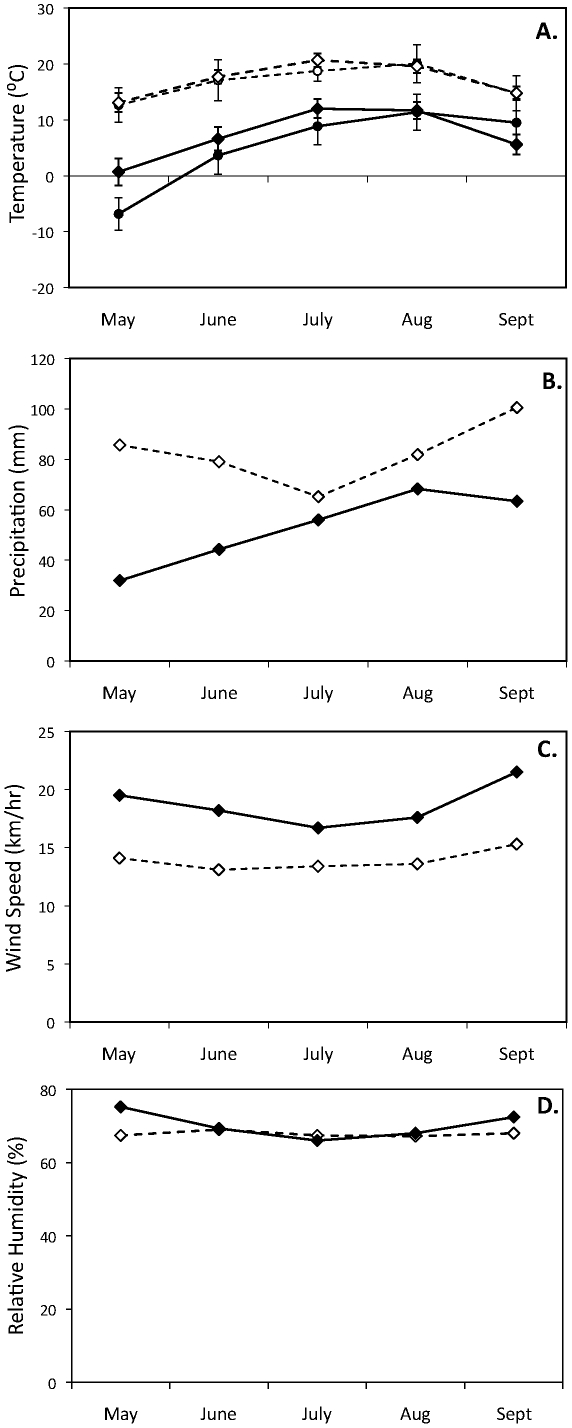
Environmental conditions during the breeding season for northern Manitoba and southeastern Ontario. Differences in A) temperature (°C), B) precipitation (mm) (includes both rain and snow), C) wind speed (km/h,) and D) relative humidity (%) between northern Manitoba (filled circles) and southeastern Ontario (un-filled circles). Circles represent averaged monthly values for 1971–2000± SD (standard deviation data only available for temperature); temperature data for 2009 are shown as triangles (all environmental data were collected from Environment Canada 2010). Northern Manitoba is typically, colder, drier and windier than southeastern Ontario, with comparable levels of humidity.

Our northern site in Churchill, Manitoba, Canada (58°40′N, 94°25′W, elevation ∼20 m asl) is located at the transition of boreal forest and tundra. Habitat includes stunted spruce (*Picea glauca* and *P. mariana*) and larch (*Larix laricinia*) trees, willow thickets, and scattered tundra flats and fens with grassy tussocks. Habitat at our southern site in Elgin, Ontario, Canada (44°34′ N, 76°19′ W, elevation ∼125 m asl) includes mixed deciduous forest with scatted marshes and waterways and small farms near forested tracks. In addition to differences in habitat, northern Manitoba is colder (Churchill: 10.1±3.0°C, Elgin: 17.2±3.2°C), drier (Churchill: 56.2±12.0 mm, Elgin: 82.5±12.7 mm), and windier (Churchill: 17.5±0.75 km/h, Elgin: 13.9±0.9 km/h) throughout the summer months (Fig. 1; all data from Environment Canada 2010 [23]). Our two sites also differ in their predator and parasite communities (C. Crossman pers. observ.).

If nest morphologies across species show similar responses to shared selective pressures across our sites (e.g., most species increase nest mass under cold conditions), then we predicted that nests in northern Manitoba would differ in similar ways from nests in southeastern Ontario. Of the six aspects of nest morphology we quantified, we specifically predicted that birds in northern Manitoba would either build heavier nests or construct thicker nest-walls, making nests better suited to colder conditions at this site [14], [18]. After assessing morphological differences in nests, we deconstructed nests of each species to analyze possible differences in nest composition. Nest composition can theoretically change in three ways. Larger nests could result from individuals adding (i) new materials, (ii) larger amounts of the same materials in equal proportions, or (iii) disproportionate amounts of select materials. Any of these possibilities are plausible and each species may adjust the construction of their nests differently. For species breeding in northern Manitoba, we predicted greater amounts of all materials, and especially materials that provide good thermal insulation [24].

## Materials and Methods

### Study species

American Robins breed throughout North America in a diverse array of habitats from cities to rural areas, and nest placement is highly variable. All of our measured and collected robin nests from northern Manitoba were placed in spruce trees near the trunk, and all but one nest from southeastern Ontario were placed in deciduous trees often near the trunk; the other was placed in a short, dead Eastern White Pine (*Pinus strobus*) near the crown. We excluded all nests placed on man-made structures. Sallabanks and James [25] report morphological measures of robin nest from five locations across the United States and Canada suggesting some geographic variation. Females typically lay 3–4 egg clutches in April through July and, in southern sites, successful breeding is often followed by a second brood [25].

Yellow Warblers are one of the most abundant and widespread warblers in North America, breeding from northern Canada to Peru [26]. Briskie [17] first described geographic variation in Yellow Warbler nests. Individuals breeding in northern Manitoba built larger, thicker nests than those breeding in southern Manitoba. Nests are often placed in upright forks of branches in short deciduous shrubs [26]. Females usually lay 4–5 egg clutches and, when early nests fail, will readily rebuild nests [26].

Savannah Sparrows breed from central Mexico north to the Arctic Circle. Unlike other species we examined, Savannah Sparrows place their nests on the ground, often in fields next to grass tussocks or at the base of forbs or shrubs; most nests have a roof-like covering of grasses and forbs [27]. Baird [28] reported similar nest construction across their range, but does not provide details of nest measurements and morphology. Females usually lay 4–5 egg clutches and will readily re-nest in mid-latitude and southern sites if previous nests are destroyed [27].

Common Redpolls breed throughout Alaska, northern Canada, the southern coast of Greenland and high latitude Eurasia. In northern Manitoba, early breeding redpolls typically place their nest on horizontal limbs of spruce trees, but later nesting individuals often nest in willow and larch trees [29]. Despite changes in nesting trees, nests usually have a base of sticks and a thick nest lining of feathers and soft plant material. All redpoll nests that we measured and collected were placed in spruce trees and all had 3–5 egg clutches.

American Goldfinches breed throughout southern Canada and much of the continental United States. Many populations of American Goldfinches are unique among other temperate breeding passerines because of their late breeding season, which at our study site in southeastern Ontario typically starts in early July but often continues into early September [30]. Females typically lay 5–6 egg clutches [31]. All goldfinch nests that we measured were placed between 0.8–1.2 m above ground in short deciduous trees often near edges of open habitats.

### Nest collection

We found nests by observing females carrying nesting material or making repeated trips to a single site, and by flushing incubating females while walking through appropriate habitat. At both study sites, we found five nests of each species and all nests were collected after natural predation events or after fledging.

### Nest morphology

Once a nest either fledged young or failed, we carefully removed the nest from its substrate and immediately placed it into a bag to prevent the loss of nesting materials. We left bags open to allow nests to air dry in the same ambient lab conditions for 1–2 months and determined the dry mass of all nests using an electronic balance.

In addition to nest mass, we measured five aspects of nest morphology: 1) external nest diameter, 2) internal nest-cup diameter, 3) external nest depth, 4) internal nest-cup depth, and 5) nest-wall thickness. Measures of the external and internal nest-cup diameter are the average of the maximum and minimum diameters of the outer and inner nest-cup, respectively. External nest depth is the distance from the top rim of the nest walls to the bottom of the nest's exterior. Internal nest-cup depth is the distance from the top rim of the nest walls to the base of the interior nest cup (where eggs are placed). Measures of nest-wall thickness represent the average of eight evenly spaced measurements of the nest wall to help account for variation in nest-wall size and shape. All measures of nest morphology that we used in our analyses were made by CAC to control for inter-observer variation, and all measures were made on nests prior to hatching or the presence of large nestlings because nestlings can distort the shape of the nest [32]. All nest dimensions were measured to the nearest 0.5 mm with a manual caliper and all nest measures for each species are summarized in Table 1. To account for possible changes in nest morphology throughout the breeding season, we only used nests constructed early in the breeding season at each study site (southeastern Ontario: all nests collected 4–28 May 2009, except American Goldfinch nests collected 26–30 July 2009, northern Manitoba: all nest collected 17 June–25 July 2009). While breeding in northern Manitoba can start in late May and early June, the spring of 2009 was one of the coldest on record, delaying breeding by nearly a month [23].

**Table 1:** Summary of nest measurements.

	American Robin		Yellow Warbler		*Carduelis*		Savannah Sparrow	
	Churchill	Elgin	Churchill	Elgin	Churchill	Elgin	Churchill	Elgin
Nest mass	246.1±45.2	163.8±40.0	9.6±2.1	5.7±1.4	16.6±3.3	9.7±2.2	8.6±3.4	10.6±3.1
Nest-wall thickness	17.3±3.6	14.0±2.8	11.9±0.9	8.1±1.0	15.9±2.7	12.0±0.5	9.2±4.6	10.8±3.0
Exterior nest diameter	124.2±4.6	113.2±4.0	71.7±3.8	63.1±1.5	78.5±6.1	73.3±1.0	83.1±9.9	79.8±10.2
Interior nest-cup diameter	93.9±4.0	86.7±6.5	49.9±1.8	48.7±1.8	49.8±3.4	48.6±1.0	70.5±6.9	60.9±2.9
Exterior nest depth	97.8±14.8	96.8±13.8	71.1±6.6	59.6±12.0	55.8±6.0	70.5±9.4	55.8±10.6	54.3±8.4
Interior nest- cup depth	59.6±5.1	50.0±3.8	31.1±4.9	33.9±2.2	29.0±2.2	33.7±4.1	33.2±4.5	43.4±2.5

Summary of nest measurements taken from nests for each species from our northern site near Churchill, Manitoba and our southern site near Elgin, Ontario. Measures of the outer and inner nest cup diameter are the average of the maximum and minimum diameters of the outer and inner cup. Outer nest depth is the distance from the top rim of the nest walls to the bottom of the nest's exterior. Inner cup depth is the distance from the top rim of the nest walls to the base of the interior nest cup (where eggs are placed). Nest dimensions are the average measurements (in g or mm) of five nests (± SD). N = 5 for all species except *Carduelis* in Elgin where N = 4.

To ensure that our field measures were consistent, we re-measured a single aspect of nest morphology, nest-wall thickness, four additional times in the laboratory on all collected nests that did not fledge young; all but three nests failed to fledge young, thus we were able to take repeated measures of nests in the lab for nearly all collected nests, with the exception of Savannah Sparrow nests. For two species where CAC was not present to measure nests, we used only nest measures made in the lab (by CAC) on unsuccessful nests that were not altered by the presence of large nestlings (*N* = 10 for Yellow Warblers and *N* = 4 for American Goldfinches). We used only field measures for Savannah Sparrow nests because, as ground nests, they changed shape dramatically after collection making it impossible to take repeated measures of these nests in the lab; unfortunately, we did not measure Savannah Sparrow nests multiple times in the field because we did not anticipate these nests losing their shape so dramatically when collected. In all cases where we measured nests multiple times, repeated measures of individual nests were highly correlated and highly significant (intraclass correlation coefficient for consistency in repeated measures of nest-wall thickness for: American Robin 0.74, *F*_8,36_ = 15.0; *Carduelis* 0.75, *F*_8,36_ = 16.2, Yellow Warbler 0.97, *F*_9,40_ = 182; *P*<0.00001 for all three groups [33]), suggesting that our nest measures for each nests were highly consistent. We used the average of all repeated measures of nest-wall thickness for each nest in our analyses.

### Nest composition

To examine possible differences in materials used in nest construction between breeding locations, we examined the composition of 10 nests of each species (5 from southeastern Ontario, 5 from northern Manitoba), with the exception of Savannah Sparrow where we could not relocate one nest after a predation event at our site in southeastern Ontario. We separated materials into 12 different categories and grouped materials that we could not identify into the category that it most closely resembled (see Table S1). Our categorization of materials allowed us to compare nest composition between sites, even though different plants are found at each location. To decide if a material should be categorized separately, we compared the average mass of each material to the average total nest mass for each species in each location. If a material constituted ≥1% of the average nest mass for a location, it was grouped in its own category; all materials with masses <1% of the average nest mass were categorized as miscellaneous material.

### Statistical analyses

To test the hypothesis that nests morphologies differed between our study sites, we used a multivariate analysis of variance (MANOVA) in R (R version 2.12 [34]) where multiple aspects of nest morphology (nest mass, nest-wall thickness, interior nest-cup diameter, exterior nest diameter, interior nest-cup depth, exterior nest depth) were our dependent variables and species and location and a species*location interaction term were our independent variables. For all aspects of nest morphology that showed significant or near significant (*P*<0.1) species*location interaction terms, we conducted post-hoc tests to test for species-specific effects on nest morphology between locations. All measures of nest morphology were log transformed to better fit the assumptions of MANOVA tests.

To test the hypothesis that nest composition differed between breeding sites, we used Chi-squared tests in R (R version 2.12 [34]) using the proportion that each material contributed to the average nest mass within a location for each species. For species with significant differences in nest composition, we also examined how the mass of various nesting materials differed between locations. We used parametric two-tailed *t*-tests for all material groups that fit a normal distribution, including some material groups that required transformations to achieve a normal distribution. Mass of feathers in *Carduelis*, and hard grasses/sticks, leaves, feathers, casings and fur/hair in Yellow Warblers, could not be normalized through transformations, so these materials were analyzed using non-parametric two-sample Mann Whitney *U*-tests [35]. If a material category was normally distributed for one location, but not the other, we used non-parametric tests to compare material categories between locations. We excluded the miscellaneous category for all analyses of nest composition.

## Results

### Nest morphology

**Figure 2:**
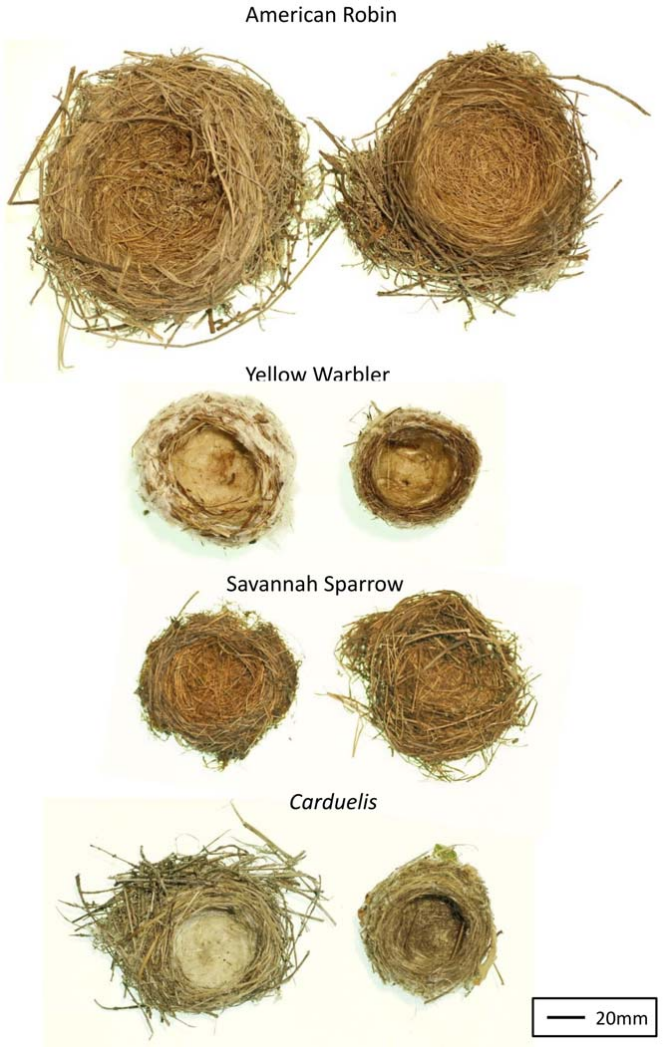
Photographs of nests illustrating visible differences in nest morphology and composition. Representative nests from northern Manitoba (left) and southeastern Ontario (right) for American Robin, Yellow Warbler, Savannah Sparrow, and *Carduelis* finches (Common Redpoll on left and American Goldfinch on right). Note that the shape and morphology of the Savannah Sparrow nests are distorted because they are ground nests that lose their structure when removed from the nest site.

Between study sites, nests were visibly different in all species except Savannah Sparrows (Fig. 2). General nest morphology among species differed between locations (MANOVA, location, *F*_1_ = 3.36, *P* = 0.014), and individual species changed their nest morphologies in different ways (MANOVA, species*location interaction term, *F*_3_ = 3.03, *P* = 0.0003). Below, we detail what aspects of nest morphology varied, and how each species differed in their nest morphologies, between locations.

**Figure 3:**
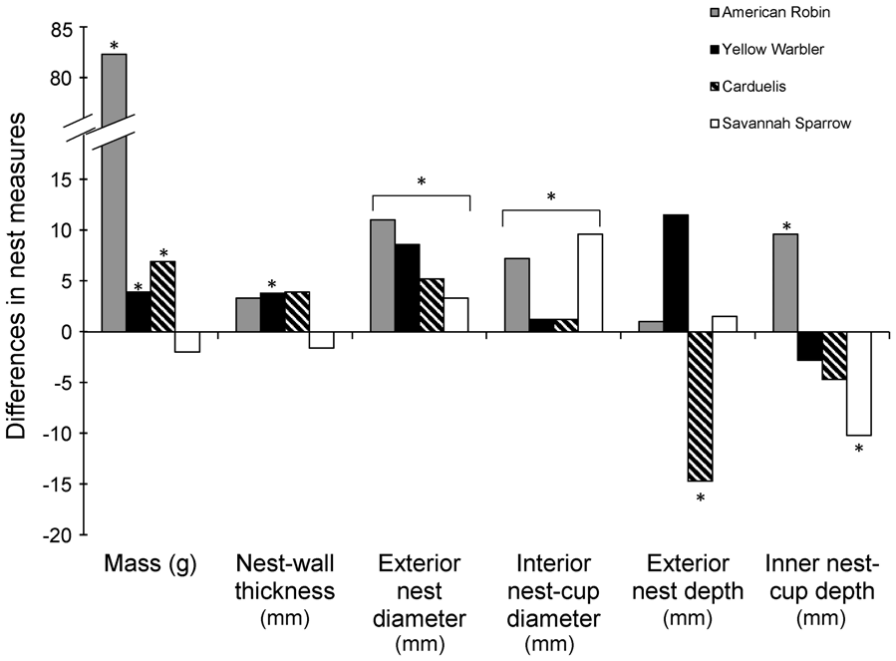
Differences between study sites in six measures of nest morphology. Differences in six measures of nest morphology between northern Manitoba and southeastern Ontario for American Robin (grey), Yellow Warbler (black), *Carduelis* (hatched), Savannah Sparrow (white). Bars represent the difference in nest morphology between sites (e.g., [nest mass in northern Manitoba]-[nest mass in southeastern Ontario]), such that positive values represent nest measures that are larger in northern Manitoba, and negative values represent nest measures that are larger in southeastern Ontario. Nest mass is recorded in grams, all other nest variables are recorded in millimeters. * denotes a significant difference (P<0.05) between study sites.

Nest masses were generally heavier for nests from northern Manitoba than nests from southeastern Ontario (*F*_1_ = 11.5, *P*<0.01, Fig. 3). However, not all species had heavier nests in northern Manitoba (species*location interaction term, *F*_3_ = 4.2, *P*<0.013). Nests of American Robins (*t* = −2.33, *P* = 0.03), Yellow Warblers (*t* = −2.70, *P* = 0.01), and *Carduelis* finches (*t* = −3.08, *P* = 0.004) were heavier in northern Manitoba. The mass of Savannah Sparrow nests did not differ between locations (*t* = 1.423, *P* = 0.17), but tended to be lighter in northern Manitoba (Fig. 3).

Nest walls were generally thicker in northern Manitoba than southeastern Ontario (*F*_1_ = 5.29, *P*<0.029, Fig. 3), but not all species had thicker nest walls in Churchill (species*location interaction: *F*_3_ = 2.78, *P* = 0.058). Nests of Yellow Warblers (*t* = −2.648, *P* = 0.01) in northern Manitoba had thicker walls than those in southeastern Ontario, and this pattern approached significance in *Carduelis* finches (*t* = −1.749, *P* = 0.09). Nest-wall thickness did not differ significantly between sites for American Robins (*t* = −1.386, *P* = 0.18) and Savannah Sparrows (*t* = 1.285, *P* = 0.21); robins showed a trend for thicker nest walls in northern Manitoba, while the sparrows showed the opposite pattern (Fig. 3).

Nests from northern Manitoba had thicker internal (*F*_1_ = 8.61, *P* = 0.0064) and external (*F*_1_ = 13.42, *P* = 0.00095) nest-cup diameters (Fig. 3), and neither measure of nest morphology showed a significant species*location interaction term (internal *P* = 0.12, external *P* = 0.71 nest diameter).

Interior nest-cup depth showed no significant differences among locations (*F*_1_ = 1.55, *P* = 0.22, Fig. 3), but showed a significant species*location interaction (*F*_3_ = 4.77, *P* = 0.0078). American Robins had deeper interior nest-cups in northern Manitoba (*t* = −2.04, *P* = 0.050), Yellow Warblers and *Carduelis* finches showed no differences in inner nest-cup depth between locations, and Savannah Sparrows showed significantly shallower inner nest depths in northern Manitoba as compared with southeastern Ontario (*t* = 3.01, *P* = 0.0052).

Exterior nest depth showed no consistent patterns between locations (*F*_1_ = 0.029, *P* = 0.87, Fig. 3), but a significant species*location interaction (*F*_3_ = 2.99, *P* = 0.047). American Robins and Savannah Sparrows showed no differences in exterior nest-depth between locations (*P*>0.6 for both species), and Yellow Warblers tended to have deeper exterior nest depths in northern Manitoba (*P* = 0.060), and *Carduelis* finches showed shallower exterior nest depths in northern Manitoba as compared to southeastern Ontario (*t* = 2.22, *P* = 0.034).

### Nest composition

**Figure 4:**
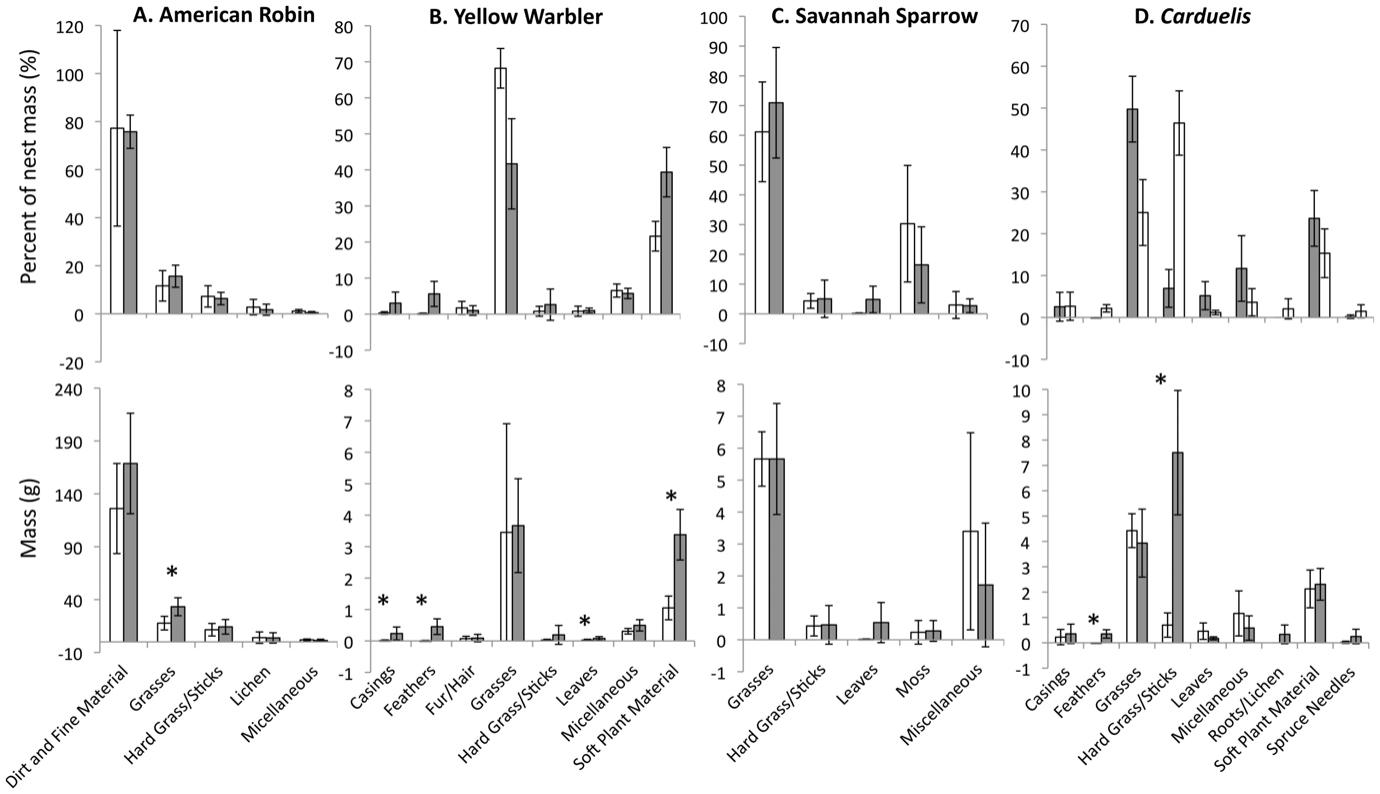
Nest composition categorized by material for nests of each species. Comparison of nest composition for each species that bred in northern Manitoba (grey bars) and southeastern Ontario (white bars). Bars represent dominant nesting materials (≥1% of nest mass). Materials that comprised <1% of nest mass are combined into the miscellaneous category. Plots show both average dry mass (g) of each material (± SD) and % of average dry nest mass (± SD) of each material category (* indicates significant differences where *P*≤0.05).

The nest composition of each species differed between locations in different ways (Fig. 4), thus we treat each species separately.

American Robins in both northern Manitoba and southeastern Ontario constructed nests almost exclusively of coarse grasses, sticks, lichens, and mud, and lined nests with dry grasses. Robins in northern Manitoba built larger, heavier nests by increasing the amount of all materials more or less equally (χ^2^_3_ = 0.9, *P* = 0.82, Fig. 4A).

Yellow Warblers in northern Manitoba built larger, thicker-walled nests than those in southeastern Ontario. Larger nests resulted from an increase in some, but not all materials (χ^2^_7_ = 22.3, *P* = 0.0023); nests from northern Manitoba had significantly more casings (*U*<0.0001, *Z* = −2.6, *P* = 0.008), feathers (*U*<0.0001, *Z* = −2.6, *P* = 0.008), leaves (*U* = 3.0, *Z* = −2.0, *P* = 0.045), and soft plant-material (*t*(8)  = 5.9, *P<*0.001). We found no differences between sites in the amount of fur/hair, grasses, and hard grasses/sticks in nests (Fig. 4B, *P*≥0.28, for all non-significant materials).

Savannah Sparrow nests in northern Manitoba and southeastern Ontario did not differ in either mass (*F*_1,7_ = 0.9, *P* = 0.38) or nest-wall thickness (*F*_1,8_ = 0.4, *P* = 0.53). In addition, we found no significant differences in the proportions of grasses, leaves, mosses, and hard grasses/sticks in nests between sites (χ^2^_3_ =  3.8, *P* = 0.29, Fig. 4C).

*Carduelis* finches in northern Manitoba had heavier nests with thicker walls, and these differences were due to an increase in some materials and not others (χ^2^_7_ = 46.6, *P*<0.001). Redpoll nests in northern Manitoba had significantly more feathers (*U*<0.0001, *Z* = −2.6, *P* = 0.008) and more hard grasses/sticks (*t*(4.3)  = 6.1, *P* = 0.003), whereas the amount of other materials did not differ between study sites (Fig. 4D).

## Discussion

We found that three phylogenetically-independent lineages showed similar patterns of variation in nest structure between locations, suggesting that populations are adapting to similar selective pressures. American Robins, Yellow Warblers, and *Carduelis* finches breeding in northern Manitoba constructed heavier nests, tended to have thicker nest-walls, and had wider interior and exterior nest diameters than conspecifics/congenerics breeding in southeastern Ontario (Fig. 3). To achieve larger nests, these three groups altered the composition of their nests in unique ways, suggesting that they use species-specific strategies for adapting to similar selective pressures.

The pattern of heavier nests and/or nests with thicker nest-walls in northern Manitoba is consistent with other studies. European birds breeding in colder, often northern regions [21], hummingbirds breeding at high elevations [36], and weaver birds breeding in colder, higher elevations [37] build larger or better insulated nests than counterparts breeding in warmer locations or lower elevations. This pattern is not exclusive to birds. Several species of nest building rodents, including *Peromyscus* mice [38], Flying Squirrels, *Glaucomys volans* [39], Hispid Cotton Rats, *Sigmodon hipidus* [40], and other species (see [41]), build larger nests when breeding in colder regions. The repeated pattern of nest size increasing with elevation and latitude strongly suggests that selective pressures common to these locations favor larger nests. While differences in climate (notably temperature) are a logical explanation, several other factors may influence nest morphology (e.g. predation, parasitism, varying reproductive investment), but these alternatives are often overlooked because data addressing these are scarce.

### Possible factors selecting for different nest morphologies

#### Climate

Colder temperatures in northern Manitoba should select for larger, thicker-walled nests because they retain heat better than small nests [14], [18]. Nests that retain heat in cold environments should reduce energy expenditure for incubating and brooding parents and help maintain warm temperatures for embryo development and nestling growth. The importance of nest temperature for parental energetic demand has been demonstrated in at least three species of birds, where females with experimentally heated nest sites expended less metabolic energy during incubation, were in better physical condition, fed nestlings more frequently, and raised young that grew at faster rates [42]–[44].

In addition to larger nest size, increasing the amount of insulative materials should help overcome the challenges of breeding in cold environments [24]. Both Yellow Warblers and Common Redpolls used more soft plant material and feathers in northern Manitoba; these materials are excellent insulators [9], [24] and further suggest that cold temperatures are a challenge common to species breeding at our site in northern Manitoba.

Differences in wind speed and precipitation between breeding sites is consistent with different nest morphologies. Windier conditions in northern Manitoba should further reduce nest temperatures through convective heat loss, thus favoring larger nests. Less precipitation should also allow species in northern Manitoba to use greater amounts of insulative materials without suffering increased energetic costs of bulky nests that absorb more water and take longer to dry [14], [18], [24]. In contrast to temperature, wind and precipitation, we found no differences in relative humidity between breeding sites (Fig. 1), suggesting that humidity should have little influence on the differences in nest morphologies that we observed.

#### Predation and brood parasitism

Many cues may signal nest locations to predators, such as scent [45], bird activity [46], and nest size [15]. If predators use visual cues to locate nests, then larger nests should be more conspicuous and suffer higher rates of predation [15]. Latitudinal patterns of nest predation suggest that predation decreases with increasing latitude [47], [48], presumably because of reduced predator diversity and abundance. Consistent with predicted latitudinal patterns of predation, our southern site has a greater diversity of potential nest predators including snakes, rodents, foxes, skunks, weasels, and other birds [49] compared with our northern site that lacks snakes [50] and chipmunks [51]. In addition, the abundance of nest predators (e.g., red squirrels, and jays) at our northern site appears lower (C. Crossman pers. observ.). These differences in the nest predator communities between our sites could result in higher nest predation rates and consequently, selection for smaller nest sizes in southeastern Ontario.

Similarly, brood parasitism from Brown-headed Cowbirds may favor smaller nests if cowbirds use visual cues to detect host nests [52]. Cowbirds are absent from northern Manitoba [53], but regularly attempt to parasitize our study species in southeastern Ontario, and thus may act as a common selective agent on nest size in southeastern Ontario. However, cowbirds are not native to our southern study site and have only recently colonized southeastern Ontario as a result of anthropogenic changes to habitat [54], therefore any adaptive response to cowbird parasitism is either recent or a result of selection occurring in other areas in the breeding range of our study species.

#### Ectoparasitism

Bird nests are often parasitized by arthropod ectoparasites such as mites, ticks, fleas, and flies [9]. Ectoparasites in nests are known to reduce growth rates and survival of nestlings [55]–[57], however, their influence on nest morphology remains largely unstudied. Comparative studies suggest that ectoparasites are more diverse and abundant at lower latitudes [47]. If species in southeastern Ontario were responding to greater intensities of ectoparasitism, then they may use more materials with antibiotic properties (e.g., green plant materials, aromatic materials) [16], or limit the amount of nesting material within which ectoparasites can hide. This alternative explanation awaits further study along with comparative work that examines ectoparasite diversity between our study sites.

#### Life Histories

Bird populations at higher latitudes typically have higher annual fecundity and lower annual survival rates than populations at lower latitudes [58], [59]. Birds in northern Manitoba may therefore allocate more energy to reproduction (e.g., lay larger clutches or invest more energy in nest construction) compared with birds in southeastern Ontario, and this difference in reproductive investment could explain the larger nests we observed in northern Manitoba. Two lines of evidence suggest that differences in life history strategies are not responsible for the variation in nest morphologies we observed. First, in all four lineages of birds, clutch sizes appear similar between locations (see Table S2), thus the larger nests in northern Manitoba are unlikely to result from larger clutch sizes. Second, if variation in life history strategies causes different nest morphologies between our sites, we would expect all species in our study to show similar patterns of larger nests in northern Manitoba. The lack of similar patterns of variation in nests of Savannah Sparrows, as well as other ground nesting species that nest at high latitudes [19], suggests that ecological factors (e.g., climate, predation) cause the observed variation in nest morphology, rather than variation in life histories.

In addition to increased reproductive investment at higher latitudes, body size often increases with cold temperatures and/or latitude (Bergmanns' rule [60], English translation [61]). Unfortunately, we do not have measures of body size for our species at each location, as we did not capture birds for this research. If large nest morphologies in northern Manitoba were the result of larger body size, we might expect dimensions of the interior nest-cup to be wider and deeper in northern Manitoba than in southeastern Ontario. Interior nest-cup diameters were generally wider in northern Manitoba (for all species combined, including Savannah Sparrows), but interior nest-cup depths were not consistently deeper (Fig. 3). While our results suggest that adult body size could contribute to the larger nest morphologies that we observed in northern Manitoba, further studies are required to test whether such an effect exists.

#### Access to nesting materials

An alternative hypothesis to explain geographic variation in nests is the differential access to nesting materials between study sites. While birds at our two study sites have access to different nesting materials, birds at both study sites have access to nesting materials with similar properties that correspond to the classification groups of materials in this study (see Table S1). Thus, birds at both sites had access to all groups of materials, but the degree of access may have differed between sites and may influence the differences in nest morphology and composition that we observed.

Among our focal species, Savannah Sparrows did not show similar patterns of variation in nest morphology and composition between study sites compared to the other species we examined; this is consistent with other accounts of geographic variation in Savannah Sparrow nests [28]. In the case of ground-nesting species like the Savannah Sparrow, the nest structure itself may be less important than the nest site as a buffer to environmental conditions [19], [20], [62]. In northern Manitoba, four of the five Savannah Sparrow nests were at the base of grassy tussocks buffering them from wind and this protected nest placement is similar to other ground-nesting sparrows that breed at our site in northern Manitoba (White-crowned Sparrow (*Zonotrichia leucophrys*) [19], American Tree Sparrow (*Spizella arborea*) C. Crossman pers. observ.).

Other alternative hypotheses could also explain why Savannah Sparrows did not show similar variation in nest morphology as above ground nesting species between our sites. Ground nesting species like the Savannah Sparrow may be especially vulnerable to terrestrial nest predators (e.g., rodents, mustelids), and these predators may exert similar selective pressures on nest morphology between our sites, resulting in less pronounced geographic variation in nest morphology (see also [63], [64]). Alternatively, if nest-building behavior has a heritable basis, Savannah Sparrows may show less geographic variation in their nests because high dispersal and gene flow impede local adaptation in nest morphology. This alternative to explain the lack of variation in nest structure in Savannah Sparrows seems less plausible, because we can think of no reason why dispersal and gene flow should differ markedly between ground- and above-ground nesting species.

That the nests of Savannah Sparrows and other ground nesting sparrows [19], [20] do not show similar patterns of variation in nest morphologies as compared to above-ground nesting species raises interesting questions about geographic variation in nests and nesting habits. Might ground-nesting species show less variable nest morphologies than above-ground nesting species? Do cavity-nesting species also show less variation in nest morphologies? Both of these hypotheses seem plausible because ground- and cavity-nests are less exposed to climatic conditions – a strong selective agent that likely influences the evolution of nest morphology.

In summary, three out of four phylogenetically-independent lineages of birds showed convergence in nest morphologies, constructing heavier, and sometimes thicker-walled nests with wider interior and exterior nest-cup diameters in northern Manitoba compared with southeastern Ontario. Convergence of nest morphologies among three independent lineages of birds suggests that differences in nest morphologies represent an adaptive response to common selective pressures that differ between breeding sites. Nests from ground-nesting Savannah Sparrows showed different patterns of variation than the above-ground nesting species we examined, thus we speculate that Savannah Sparrows may be subject to different selection pressures. Species with larger nests in northern Manitoba adjusted the composition of their nests in different ways – American Robins used greater amounts of all materials, while Yellow Warblers and *Carduelis* finches used greater amounts of insulative materials. Together, our findings suggest that these species are adapting to similar selective pressures within breeding sites in slightly different ways.

## Supporting Information

Table S1 **Description of material categories for deconstructed nests.**
(DOCX)

Table S2 **Average clutch size.** Average clutch size (± SD) for each species from our sites in northern Manitoba (near Churchill) and southeastern Ontario (near Elgin). Clutch size data were collected from the same nests we collected for this study. Some nests were depredated during egg laying so sample sizes vary for each species. Additional goldfinch nests found in 2010 in southeastern Ontario, but not used in this study, were also included to increase sample size to *N* = 4.
(DOCX)
