# Supplementary material for: Variation in the Structure of Bird Nests between Northern Manitoba and Southeastern Ontario
Source: PLoS One. 2011 Apr 28;6(4):e19086. doi: 10.1371/journal.pone.0019086 (PMC3084263; doi:10.1371/journal.pone.0019086)
Supplement: Table S1 — Description of material categories for deconstructed nests. (DOCX) [file pone.0019086.s001.docx]

Supplementary Material

**Table S1** – Description of material categories for deconstructed nests.

| **Material category** | **Description** |
| --- | --- |
| Casings | Seed casings or other small, hardened vegetation that held soft plant material or seeds. |
| Dirt/fine material | Soil or fine, silt-like dust. These materials were common in American Robin nests because of the mud nest-cup this species constructs. |
| Feathers | Feathers (including down feathers) that were actively incorporated into the nest. Stray feathers of attending females were excluded. Feathers from Willow Ptarmigan (*Lagopus lagopus*) and waterfowl were frequently used in nests from in northern Manitoba. |
| Fur/hair | Strands of animal fur or hair. |
| Grasses | Fine, thin grasses, forbs or pieces of vegetation that were flexible. |
| Hard grasses/sticks | Thick grasses, forbs, or pieces of small sticks from branches. All these materials were stiffer and more ridged than grasses. These materials were typical of American Robin and Common Redpoll nests. |
| Leaves | Fresh, dead, or dried leaves. |
| Roots and lichens | Various types of plant roots and lichens; these materials were typically fine and grass-like, but used in much lower quantities. |
| Moss | Any moss, usually flexible, green in color, and short in length. |
| Soft plant material | Soft ‘cotton-like’ plant material (e.g. thistle, willow, and cattail pappus). |
| Spruce needles | Needles from spruce trees. |
| Miscellaneous | Any and all remaining material that (i) could not be accurately identified and (ii) made up <1% of the nest weight. |
