# Supplementary material for: Variation in the Structure of Bird Nests between Northern Manitoba and Southeastern Ontario
Source: PLoS One. 2011 Apr 28;6(4):e19086. doi: 10.1371/journal.pone.0019086 (PMC3084263; doi:10.1371/journal.pone.0019086)
Supplement: Table S2 — Average clutch size. Average clutch size (± SD) for each species from our sites in northern Manitoba (near Churchill) and southeastern Ontario (near Elgin). Clutch size data were collected from the same nests we collected for this study. Some nests were depredated during egg laying so sample sizes vary for each species. Additional goldfinch nests found in 2010 in southeastern Ontario, but not used in this study, were also included to increase sample size to N = 4. (DOCX) [file pone.0019086.s002.docx]

**Table S2** – Average clutch size

|  | **northern Manitoba** | **southeastern Ontario** |
| --- | --- | --- |
| American Robin | 3.0±1.0 (*N =* 3) | 3.25±1.0 (*N =* 4) |
| Yellow Warbler | 4.0±0.0 (*N =* 5) | 4.75±0.5 (*N =* 4) |
| *Carduelis* | 4.0±0.8 (*N =* 4) | 5.0±0.8 (*N =* 4) |
| Savannah Sparrow | 4.0±1.4 (*N =* 5) | 4.0±0.7 (*N =* 5) |

Average clutch size (± SD) for each species from our sites in northern Manitoba (near Churchill) and southeastern Ontario (near Elgin). Clutch size data were collected from the same nests we collected for this study. Some nests were depredated during egg laying so sample sizes vary for each species. Additional goldfinch nests found in 2010 in southeastern Ontario, but not used in this study, were also included to increase sample size to *N* = 4
